# Impact of an air bubble within the syringe on test results obtained with a modern blood gas analyzer

**DOI:** 10.5937/jomb0-49870

**Published:** 2024-09-06

**Authors:** Laura Pighi, Gian Luca Salvagno, Roberta Ferraro, Giovanni Celegon, Brandon M. Henry, Giuseppe Lippi

**Affiliations:** 1 University of Verona, Section of Clinical Biochemistry and School of Medicine, Verona, Italy; 2 Cincinnati Children's Hospital Medical Center, Division of Nephrology and Hypertension, Clinical Laboratory, Cincinnati, OH, USA

**Keywords:** blood gas analysis, errors, syringe, bubble, air, gasna analiza krvi, greške, špric, mehur, vazduh

## Abstract

**Background:**

Minimizing air aspiration by carefully filling blood gas syringes is crucial to prevent air contamination from causing undesirable variations in gasses and other molecules. While some previous studies investigated this aspect, these are now outdated and only analyzed a limited number of blood gas parameters. Thus, we investigated the effects air contamination in the syringe using a modern blood gas analyzer.

**Methods:**

We sampled venous blood from 17 laboratory workers (mean age: 46±11 years; 10 women), filling two consecutive blood gas syringes. The first was filled exactly to its nominal volume (i.e., 1.0 mL), while the second was filled with 0.8 mL of blood and 0.2 mL of ambient air. Blood gas analysis was performed in each syringe using an identical analyzer.

**Results:**

In the syringe with the air bubble, we found statistically significant increase in pH (0.1%), pO2 (10.8%), SO2 (11.2%), total hemoglobin (3.0%), and hematocrit (2.7%), while values of pCO2 (-4.8%), sodium (-0.5%), and ionized calcium (-1.3%) were significantly reduced. With exception of pH, all these changes exceeded the performance specifications. Potassium, chloride, glucose, lactate, COHb and MetHb values remained unchanged.

**Conclusions:**

These findings confirm that air bubbles must be removed as soon as possible after sampling from blood gas syringes to prevent artifactual test results and misleading clinical judgment and inappropriate treatment. When blood gas syringes are received in the laboratory with air bubbles inside, the most vulnerable parameters (i.e., pO2, SO2, pCO2, sodium, ionized calcium, hematocrit and hemoglobin) should be suppressed.

## Abstract (sr)

**Uvod:**

Minimiziranje aspiracije vazduha pažljivim punjenjem špriceva za gas od krvi je ključno za sprečavanje kontaminacije vazduha da izazove neželjene varijacije u gasovima i drugim molekulima. Dok su neke prethodne studije istraživale ovaj aspekt, one su sada zastarele i analizirale su samo ograničen broj parametara gasova u krvi. Stoga smo istražili efekte kontaminacije vazduha u špricu koristeći savremeni gasni analizator krvi.

**Metode:**

Uzorkovali smo vensku krv od 17 laboratorijskih radnika (srednja starost: 46±11 godina; 10 žena), puneći dva uzastopna gasna šprica za krv. Prvi je napunjen tačno do svoje nominalne zapremine (tj. 1,0 mL), dok je drugi napunjen sa 0,8 mL krvi i 0,2 mL ambijentalnog vazduha. Analiza gasa krvi je obavljena u svakom špricu korišćenjem identičnog analizatora.

**Rezultati:**

U špricu sa vazdušnim mehurićem utvrđeno je statistički značajno povećanje pH (0,1%), pO2 (10,8%), SO2 (11,2%), ukupnog hemoglobina (3,0%) i hematokrita (2,7%), dok su vrednosti pCO2 (-4,8%), natrijum (-0,5%) i jonizovani kalcijum (-1,3%) su značajno smanjeni. Izuzev pH vrednosti, sve ove promene su premašile specifikacije performansi. Vrednosti kalijuma, hlorida, glukoze, laktata, COHb i MetHb su ostale nepromenjene.

**Zaključak:**

Ovi nalazi potvrđuju da se vazdušni mehurići moraju ukloniti što je pre moguće nakon uzorkovanja iz špriceva za gasove krvi da bi se sprečili artefaktički rezultati testova i pogrešna klinička procena i neodgovarajući tretman. Kada se u laboratoriji primaju špricevi za gas sa mehurićima vazduha, najugroženije parametre (tj. pO2, SO2, pCO2, natrijum, jonizovani kalcijum, hematokrit i hemoglobin) treba potisnuti.

## Introduction

Blood gas analysis is an important diagnostic test principally used to assess a patient's respiratory and metabolic status by measuring various parameters in anticoagulated whole blood [1]. This type of testing is typically performed in acute care or shortterm facilities such as emergency departments, intensive care units or operating rooms, to rapidly provide information on respiratory distress, shock or other severe metabolic disorders, but also in central laboratories where samples are shipped from regular wards for monitoring deterioration of acid-base balance or respiratory function [2]. The results of blood gas analysis allow clinicians to make rapid and accurate decisions about patient management, particularly oxygen therapy and fluid resuscitation. However, the correct interpretation of test results of blood gas analysis requires not only a comprehensive understanding of the numerous and complicated processes that determine variations in acid-base balance and in some other analytes measured by modern blood gas analyzers, but also a high level of quality throughout the process of collecting and analyzing patient samples [3].

As with conventional laboratory testing, errors in blood gas analysis can occur at any step of the total testing procedure (i.e., preanalytical, analytical, and postanalytical) [4], and can significantly affect the accuracy and reliability of test results, potentially leading to misinterpretation of data and incorrect clinical management. Although preanalytical errors can typically be grouped into a few discrete categories involving patient and sample identification, specimen collection, management, transportation and preparation for testing (i.e., centrifugation, separation, aliquoting, etc.) and storage, the syringes used for blood gas testing are only susceptible to the first parts of errors of the total testing cycle, as the test is performed with whole blood and does not require specific activities for transportation, preparation and storage, except when the testing site (i.e., the central laboratory) is distant from the site for collection (i.e., the clinical ward) [5]. Therefore, the more common preanalytical errors in blood gas analysis include identification errors, inappropriate sample collection (e.g. incorrect syringe filling), contamination with other exogenous fluids, air exposure (and incorporation), inappropriate management (e.g., inaccurate mixing), clotting, hemolysis, and incorrect storage time and tempe rature when bedside testing is not possible, and samples need to be transported to another testing site [6] [7].

Previous studies have reported that the contact of sample with (ambient) air and incorporation of air bubbles into the diagnostic sample (i.e., the blood gas syringe) can lead to significant changes in the concentration of some gasses and molecules measured by blood gas analyzers [8] [9] [10] [11] [12], thus compromising measurement accuracy when the air is not removed from the syringe immediately after sampling, as also clearly highlighted in the Clinical and Laboratory Standards Institute (CLSI) C46A2 approved guideline for blood gas analysis [13]. This is a serious problem, in that the presence of air bubbles in blood gas syringes has been reported as high as 14% of all specimens [14]. Since most previous studies on this topic are outdated and limited to a relatively small number of parameters [8] [9] [10] [11] [12], we examine here the effects of the presence of an air bubble in the blood gas syringe on the results of several conventional and innovative parameters measured with a modern blood gas analyzer.

## Materials and methods

**Figure 1:**
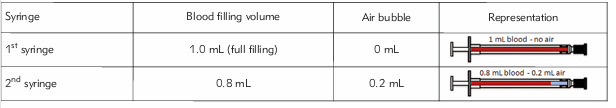
Graphical description of the study evaluating the impact of an air bubble within the syringe on test results of blood gas analysis.

We collected venous blood from 17 laboratory workers (mean age; 46±11 years; 10 women) employed in the Service of Laboratory Medicine of the Academic Hospital of Verona (Italy). An accessible vein in one of the upper arms was punctured using a 21G × 3/4” (0.8×20 mm) butterfly device (Safety Blood Collection Set, Gemtier Medical, Shangai, China), to which an evacuated 3.5 mL lithium heparin blood tube (Vacutest Kima, Padova, Italy) was first connected to remove the dead space inside the tube. Immediately thereafter, venous blood was manually aspirated within two heparinized 1.0 mL, 0.5 mm × 16 mm blood gas syringes (Arterial Blood Sampling Kit, Smiths Medical ASD IN, Minneapolis, MN, USA). The first syringe was filled exactly to its nominal volume (i.e., 1.0 mL), while the second was filled with 0.8 mL of venous blood and the remaining empty space in the 1.0 mL syringe was then filled by aspirating 0.2 mL of room/ambient air, which typically contains 78% nitrogen and 21% oxygen and minute amounts of carbon, helium, methane, argon and hydrogen (Figure 1). This specific experimental design was established because the majority of blood gas syringes contaminated with air bubbles received in the local laboratory have air bubbles (i.e., deaths space) of around 0.1–0.3 mL. The syringes were capped immediately after collection and mixed by rotation between the palms of the hands for around 20 sec, thus ensuring an accurate mix between the additive (i.e., lithium-heparin) and venous blood (and ambient air, in the case of syringes containing the death space).

Blood gas analyses of all syringes (manually transported) were always performed between 15 min after sampling, with an identical analyzer and the same test cassette (GEM Premier 5000, Instru mentation Laboratory, Monza, Italy). Before the test, 0.2 mL of venous blood were removed from the first collected syringe (i.e., that completely filled with 1.0 mL of venous blood), while the 0.2 mL of air were removed from the second drawn syringe (i.e., that containing 0.8 mL of blood plus 0.2 mL of ambient air), so that both syringes contained an identical final volume of venous blood for testing. The results of the blood gas analysis were expressed as mean and standard deviation (SD). The significance of bias obtained between the reference syringe completely filled with 1.0 mL of venous blood and that containing 0.8 mL of venous blood and 0.2 mL of ambient air was defined as percent variation exceeding the performance specifications propositioned by Kuster et al. [15], as summarized in Table 1. Variations of analyte concentrations between the two paired syringes were evaluated with paired-sample Student’s T test, Spearman’s correlation, while the relative bias was assessed using Bland and Altman plot analysis. Statistical significance was set at p<0.05. The statistical analysis was performed using Analyse-it (Analyse-it Software Ltd, Leeds, UK).

**Table 1:** Impact of an air bubble within the syringe on test results of blood gas analysis (n=17). Results are presented with mean and standard deviation (SD), or mean and 95%CI (95% confidence interval), when appropriate. Biases beyond performance specification for each analyte are reported in bold font. ^1^Intra-assay imprecision locally calculated on 10 runs ^2^Compared to the reference full syringe<br>95%CI, 95% confidence interval; pCO_2_, partial pressure of carbon dioxide; pO_2_, partial oxygen pressure; sO_2_, oxygen saturation; iCa^2+^, ionized calcium; Glu, glucose; Lac, lactate; Hct, hematocrit; tHb, total hemoglobin; COHb, carboxyhemoglobin; MetHb, methemoglobin.

Analyte	Performance specification	Intra-assay imprecision^1^	1.0 mL full syringe	1.0 mL syringe with (0.2 mL) air bubble		
			Value	Value	P-value2	Bias (95%CI)*
pH	±1.5%	±0.1%	7.37±0.03	7.38±0.03	0.006	0.1% (0.0% to 0.2%)
pCO_2_ (mmHg)	±2.4%	±1.4%	47.8±5.5	45.6±5.3	<0.001	-4.8% (-6.5% to -3.1%)
pO_2_ (mmHg)	±1.5%	±1.7%	38.8±16.8	43.8±19.5	0.006	10.8% (5.4% to 16.3%)
sO_2_ (%)	±1.5%	±1.2%	55.4±22.6	61.8±23.4	0.001	11.2% (5.3% to 17.1%)
Sodium (mmol/L)	±0.3%	±0.3%	137.3±1.4	136.6±1.2	<0.001	-0.5% (-0.7% to -0.3%)
Potassium (mmol/L)	±2.3%	±1.0%	4.26±0.26	4.19±0.22	0.069	–
Chloride (mmol/L)	±0.6%	±0.4%	104.1±1.9	104.4±1.8	0.236	–
iCa^2+^ (mmol/L)	±0.9%	±1.4%	1.25±0.05	1.23±0.05	0.006	-1.3% (-2.2% to -0.4%)
Glu (mmol/L)	±2.8%	±2.5%	5.63±0.96	5.55±0.96	0.054	–
Lac (mmol/L)	±13.6%	±7.5%	1.13±0.05	1.14±0.35	0.496	–
Hct	±1.4%	±3.6%	42.4±5.2	43.6±4.4	0.041	3.0% (0.1% to 5.9%)
tHb (g/L)	±1.4%	±2.0%	138±17	142±16	0.039	2.7% (0.0% to 5.5%)
COHb (%)	±7.5%	±43.7%	1.28±0.95	1.44±1.01	0.171	–
MetHb (%)	±11.3%	±9.3%	0.64±0.21	0.72±0.18	0.110	

All subjects recruited for this study gave written informed consent. The investigation was conducted in accordance with the Declaration of Helsinki and the relevant local legislation. The study was cleared by the Ethics Committee of the Hospital of Verona (approval number: 970CESC; July 20, 2016).

## Results

The results of this investigation are shown in Table 1. In the syringe with the 0.2 mL air bubble, a statistically significant increase in pH, partial pressure of oxygen (pO_2_), oxygen saturation (SO_2_), total hemo globin (tHb) and hematocrit (Hct) was observed, while the values of partial pressure of carbon dioxide (pCO_2_), sodium and ionized calcium (iCa^2+^) were significantly reduced. With the exception of pH, all these changes exceeded the performance specifications (Table 1). In contrast, no statistically significant changes were observed for potassium, chloride, glucose, lactate, carboxyhemoglobin (COHb) and methemoglobin (MetHb). A significant correlation between the baseline value in the full 1.0 mL syringe and the change recorded in the paired syringe with the air bubble was only found for sodium and hematocrit (Table 2).

**Table 2:** Spearman’s correlation between the baseline value of blood gas parameters in a full 1.0 mL syringe and variation recorded in a paired 1.0 mL syringe containing 0.8 mL of homologous blood and 0.2 mL of ambient air (i.e., air bubble) (n=17). Results are only presented for analytes significant variations (presented in Table 1). 95%CI, 95% confidence interval; pCO_2_, partial pressure of carbon dioxide; pO_2_, partial oxygen pressure; sO_2_, oxygen saturation; iCa^2+^, ionized calcium; Hct, hematocrit; tHb, total hemoglobin.

Analyte	Absolute variation	Spearman’s Correlation (with relative p-value)
pH	0.01 (95%CI, 0.00 to 0.01)	r=-0.43 (95%CI, -0.75 to 0.07); p=0.080
pCO_2_ (mmHg)	-2.2 (95%CI, -2.9 to -1.5)	r=-0.11 (95%CI, -0.56 to 0.39); p=0.662
pO_2_ (mmHg)	5.0 (95%CI, 1.6 to 8.4)	r=0.42 (95%CI, -0.08 to 0.75); p=0.096
sO_2_ (%)	6.5 (95%CI, 3.2 to 9.8)	r=0.22 (95%CI, -0.29 to 0.64); p=0.390
Sodium (mmol/L)	-0.7 (95%CI, -1.0 to -0.4)	r=0.91 (95%CI, 0.75 to 0.97); p<0.001
iCa^2+^ (mmol/L)	-0.02 (95%CI, -0.03 to -0.01)	r=-0.01 (95%CI, -0.49 to 0.47); p=0.977
Hct	1.2 (95%CI, 0.10 to 2.3)	r=-0.59 (95%CI, -0.83 to -0.15); p=0.013
tHb (g/L)	3.7 (95%CI, 0.2 to 7.2)	r=-0.19 (95%CI, -0.62 to 0.32); p=0.461

## Discussion

The collection of whole blood samples for blood gas analysis is a specialized procedure that necessitates technical expertise and a thorough understanding of all potential preanalytical variables that may affect the reliability of test results. To this end, there are now several lines of evidence confirming that minimizing air aspiration by carefully filling the blood gas syringe and avoiding unnecessary agitation is critical to prevent air contamination, which may cause unwarranted variations in analyte readings with blood gas analyzers. Regarding the availability of previous information on this important preanalytical aspect, the CLSI states that sample exposure to ambient (room) air can considerably impair the assessment of pH, pO_2_ and pCO_2_ due to direct contamination, as well as the concentration of iCa^2+^ due to increase binding to plasma proteins [13]. These conclusions were mostly based on previous studies, published more than 10–15 years ago, which have investigated this aspect with relatively dated instrumentation and measuring a limited number of blood gas parameters.

The first article on this important preanalytical aspect in blood gas analysis was published by Madiedo et al. in 1980 [8]. The authors first collected arterial blood into a disposable plastic syringe containing sodium heparin, and then introduced an air bubble equal to about 10% of the total volume of blood. Syringes were gently mixed, placed in ice for 15–20 min, and blood gases were measured using a Radiometer ABL-1 blood gas analyzer. The pO_2_ in these samples displayed a significant mean increase of 11 mmHg (range 1.7 to 29 mmHg), which was also directly associated with the initial value of pO_2_. 

In a second study, Biswas et al. [9] collected venous and arterial blood samples using a blood gas syringe containing lithium-heparin. In some of these specimens, the authors introduced ambient air in the syringe (0.1 of air in a 2.0 mL syringe), representing 5% of the total syringe volume. Air bubbles were left in the syringe for 1–5 minutes before being expelled and the sample being tested on a Corning analyzer. Importantly, the value of pO_2_ tended to increase after 2 min of incubation of blood with air, while that of pCO_2_ displayed an inverse trend (i.e., decrease) after 3 min of incubation of blood with air, while the pH was non-significantly affected. The maximum variation after 5 min of incubation of air with blood was +10% for pO_2_ and -9% for pCO_2_, respectively.

In 1996, Astles et al. [10] conveyed a number of blood gas syringes with a broad range of pO_2_ values through a pneumatic transport system to determine the effect of air contamination during transportation (the exact volume of air is not reported in the available text of the article). Overall, pO_2_ values increase substantially after transportation, up to 160 mm Hg. The authors also reported that blood gas syringes collected from hypoxemic patients underwent pO_2_ variations that might have triggered clinical misinterpretation (i.e., 50% of samples with baseline pO_2_ <85 mm Hg displayed increases of 10 mm Hg when contaminated with air). 

Lu et al. [11] published another interesting article in 2003. The authors filled 10 mL heparinized polypropylene syringes with pooled blood and varying volumes of ambient air (0.05 mL, 0.1 mL, 0.5 mL, and 1.0 mL, representing 0.5%, 1%, 5%, and 10% air contamination, respectively). The measurement performed on a Radiometer ABL520 evidenced a direct association between the volume of air introduced into the syringe and the increase of pO_2_ in the test sample. In syringes with 10% air contamination, the pO_2_ value increased by 24.2±3.4 mm Hg and 64.9±8.0 mmHg when conveyed to the testing site manually or by pneumatic transport system, respectively.

More recently, in 2011, O’Connor et al. [12] collected ten standard 1.0 mL blood gas syringes from 5 patients (two from each). Five syringes were left untreated while the other five were contaminated with 0.2 mL of room air. The results of testing conducted between 30–180 min on a Roche AVL OMNI3 blood gas analyzer revealed a time-dependent increase in pO_2_ values in all air-contaminated samples, accompanied by a slight decline of pH. Expectedly, the values of pCO_2_ were also significantly lower at most time points in the air-contaminated samples.

According to our protocol, encompassing a 20% contamination of ambient air in a 1.0 mL blood gas syringe containing venous blood, and with blood gas analysis performed 15 min after sampling, a number of parameters that can be assayed with modern blood gas analyzers could be biased by the presence of air bubbles in the blood gas syringe. In agreement with previous data, we confirm that pO_2_ and SO_2_ increase significantly above the clinically significant deviation threshold, while pCO_2_ shows an opposite trend. Because venous blood was used, the bias observed for pO_2_ and SO_2_ must be interpreted with caution, since their values are consistently lower than the reference ranges that one would expect for arterial blood. In addition to the findings published in other studies, we have also shown that sodium and iCa^2+^ also decreases and exceed the clinically significant variation threshold when an air bubble is present in the syringe, while hematocrit and hemoglobin increase above their respective clinically significant variation thresholds (Table 1). In most cases, with exception of sodium and Hct, we found no significant correlation between the value of the measured parameter in the fully filled syringe and the absolute change of the same analyte in the syringe containing the air bubble (Table 2). This means that, even if the data obtained in this study are tightly clustered, the bias in venous blood could be considered largely unpredictable, thus precluding the possibility of “»adjusting« the value of most parameters in the aircontaminated blood gas syringe.

Although the laboratory technology beyond blood gas analysis has not undergone substantial revolutions during the past decade, the novel generation of analyzer used in this study has never been tested before for this preanalytical problem. In summary, the evidence emerged from our study confirms that air bubbles from blood gas syringes must be removed as soon as possible after sampling to prevent artifactual test results and misleading clinical judgment and inappropriate treatment. The syringe should be inverted two or three times to check for the presence of air bubbles, which should then be expelled as quickly as possible by gently tapping one side of the syringe to bring the air bubbles to the top, and then applying light pressure to the plunger until all leftover air has been removed. 

Nevertheless, when blood gas syringes are received in the laboratory with large volume of air bubbles inside (e.g., 20% of the filling volume), the most vulnerable parameters (i.e., pO_2_, SO_2_, pCO_2_, sodium, iCa^2+^, hematocrit and hemoglobin) should be suppressed. As limitations in this study, we recognize that our results refer to a limited sample sized and to venous blood. However, this approach was chosen to avoid injury and discomfort in ostensibly healthy volunteers as typically caused by arterial punctures. Moreover, this is the very first study that has thoughtfully described the potential variation induced by ambient air contamination on a vast array of blood gas analysis parameters.

## Dodatak

### Conflict of interest statement

All the authors declare that they have no conflict of interest in this work.
